# Effects of disorganization of retinal inner layers for Idiopathic epiretinal membrane surgery: the surgical status and prognosis

**DOI:** 10.1186/s12886-023-02856-x

**Published:** 2023-03-17

**Authors:** Huanhuan Li, Conghui Zhang, Hui Li, Shuai Yang, Yao Liu, Fang Wang

**Affiliations:** 1 Department of Ophthalmology, The First People’s Hospital of Changzhou, The Third Affiliated Hospital of Suzhou University, Chang Zhou, 213003 China; 2 Department of Ophthalmology, Shanghai Tenth People’s Hospital, Tongji University, Shang Hai, 200072 China; 3 Purui Eye Group, Shanghai Purui Eye Hospital, Shang Hai, 200336 China

**Keywords:** Idiopathic epiretinal membrane, Disorganization of retinal inner layers, Surgery, Visual acuity, Optical coherence tomography angiography

## Abstract

**Background:**

To compare the surgical status in idiopathic epiretinal membrane (IERM) patients with or without disorganization of retinal inner layers (DRIL) and to correlate with optical coherence tomography angiography (OCTA) and clinical data.

**Methods:**

In 74 eyes from 74 patients with IERM treated by surgery with 12-month follow-up. According to the superficial hemorrhage, the patients were divided into group A (no macular bleeding), group B (macular parafoveal bleeding) and group C (macular foveal bleeding). Optical coherence tomography (OCT) were evaluated for presence of DRIL,central retina thickness and integrity of the inner/outer segment layer recorded at baseline and at 1, 3, 6, and 12 months postoperatively and best-corrected visual acuity (BCVA) was recorded simultaneously. OCTA was conducted at 12 months postoperatively. Main outcome measures is correlation between DRIL and superficial hemorrhage in membrane peeling,and BCVA and OCTA outcomes postoperatively.

**Results:**

The rate of DRIL and BCVA had statistically significant differences between the three groups at the time points(baseline and 1, 3, 6, and 12 months after surgery), respectively (*P* < 0.001 for all). FD-300 value (*P* = 0.001)and DCP in all parafoveal regions (superior: *P* = 0.001; inferior: *P* = 0.002;Nasal: *P* = 0.014;Tempo: *P* = 0.004) in eyes with DRIL were lower than those without DRIL.There was a linear regression relationship between FD-300 and postoperative BCVA (*P* = 0.011).

**Conclusion:**

IERM Patients with DRIL have more intraoperative adverse events and limited benefits from surgery which should be considered in the decision whether to perform mebrane peeling.OCT-A provides more detailed vascular information that extends our understanding of persistent DRIL.

## Background

Idiopathic epiretinal membrane (IERM) is a fibrous hyperplastic membrane formed in front of the macula, which can cause severe vision loss and metamorphopsia of patients. The latest researches suggested that the incidence of IERM in the people over 70 years old is as high as 15.1% [1–3].Pars plana vitrectomy (PPV) remains the predominant treatment for the IERM patients, and most physicians tend to peeling the internal limiting membrane (ILM) simultaneously [4] to avoid the recurrence of IERM [5]. Although the technique of membrane stripping is more and more stable, the intraoperative situations during membrane peeling and the recovery of postoperative visual function are quite different. A large number of clinical studies have revealed that there are no clearly indicators to accurately indicate the surgical status and prognosis of patients [3].

In the previous study, we have documented the intraoperative situations and explored the relationship between them and postoperative visual recovery. The results showed that superficial hemorrhage was the most important risk factor for postoperative poor visual recovery [6].However, the reason is still not clear yet.In 2014, Sun and associates firstly discovered the disorganization of retinal inner layers (DRIL) in optical coherence tomography (OCT) imaging [7]. DRIL was identified as a new marker for the prognosis of macular edema in various diseases, as diabetic retinopathy (DR) [7–10], and retinal vein occlusion (RVO) [11–13]. Moreover, studies about the correlation between DRIL and the prognosis of IERM surgery have attract wide attention [14].

The present study retrospectively analyzed the OCT of 74 eyes from 74 patients with IERM, which combined with the optical coherence tomography angiography (OCTA), to investigate the correlation between the structural status of the inner retinal layer and the abnormal intraoperative situations during IERM and ILM peeling,with the purpose of clarify the reasons.

## Methods

### Patient information

A total of 135 patients with epiretinal membrane peeling surgery for IERM in Shanghai 10th People's Hospital Affiliated to Tongji University (Shanghai,China) from January 2017 to January 2020.The inclusion criteria were as follows: Confirmation of ERM using funduscopic examination under slit-lamp microscopy and OCT; visual impairment; metamorphopsia;Amsler Grid Test-positive; intact outer layers of the retina at 12 months postoperative; the thickness of the central macular retina with a diameter of 1 mm (CRT-1 mm) ranged from 219 to 363 μm at 12 months postoperative (The lowest CRT-1 mm was obtained from a large sample study by using 4000 SD-OCT and the highest was the mean CRT-1 mm of 135 patients at 12 months postoperative).The exclusion criteria were as follows: history of other ocular diseases;surgery and trauma;myopia ≤ -6 diopters or hyperopia ≥  + 6 diopters;axial length ≥ 26 mm or ≤ 22 mm; occurrence of diabetes, renal dysfunction and other systemic diseases, which may have interfered with the measurements. Finally, 74 patients (74 eyes) met the criteria for inclusion and were performed a complete 12-months follow-up period.The present study followed the Declaration of Helsinki and was approved by the Institutional Ethical Review Board of Shanghai 10th People's Hospital affiliated to Tongji University (Shanghai, China).Written informed consent was obtained from all patients or their guardians at the beginning of the study.

### Surgical methods

The standard 23G three-port vitrectomy was performed under indirect panretinoscopy. In brief, triamcinolone acetonide (0.05 ml, 10 mg/ml) was injected above the optic disc using an ultra-wide-angle lens in order to identify the posterior vitreous cortex before the disc and macula, prior to performing vitrectomy.The complete detachment of posterior vitreous cortex and the posterior retinal pole was validated. Subsequently, the intraocular perfusion pressure was decreased and staining with 0.025% indocyanine green was performed for 30 s. The ILM and ERM within the macular area were peeled.All affected eyes were received combined cataract surgery and all surgeries were performed by the experienced physician.

The entire surgical process was video-recorded.The patients were classified according to superficial hemorrhage during membrane peeling. If the patients were not bleeding in the surgery, they would be put into group A. If the patients were bleeding in the area that outside the center of the macula with a diameter of 3 mm, they would be classified into group B.And, the patients with bleeding in the area that within the center of the macula with a diameter of 3 mm were classified as group C.

### Preoperative and postoperative examinations

In addition to the patients' medical history, the following information was recorded preoperatively: Sex, age, disease duration (the time of distorted vision complained by patients), best-corrected visual acuity (BCVA), Amsler grid, fundus photography (Model CR-2, Canon, Inc.) and OCT (Zeiss Cirrus HD-OCT 400). All patients were returned for follow-up at 1, 3, 6 and 12 months postoperatively. During each follow-up, the preoperative examinations were repeated. OCTA (Optovue RTVue XR Avanti, Optovue, Inc.) was performed at 12 months postoperatively.

DRIL was defined as disorganizations of the inner retinal layers, more precisely, as the inability to identify that by SD-OCT the well-known-delineated boundaries between the ganglion cell-inner plexiform layer, inner nuclear layer and outer plexiform layer within the central 1500 μm region by SD-OCT [8, 9, 15].All OCT images were interpretated by two experienced technicians.In case of different opinions, the third technician was referred to interpret the results.

OCT-A scanning was conducted under Angio Retina mode (6 × 6 mm). After acquisition of the image, the foveal region was defined as the circular region with a central diameter of 1 mm, and the parafoveal region (upper, lower, temporal, and nasal) was defined as the circular region with an outer diameter of 3 mm and an inner diameter of 1mm [9]. The software automatically segmented the tissue into 4 layers, and two of these layers were used in the following measurements. The superficial retinal layer starts from the inner limiting membrane (with an offset of 0 μm) to the inner plexiform layer with an offset of − 9 μm. The deep retinal layer starts from inner plexiform layer with an offset of − 9 μm to the outer plexiform layer with an offset of 9 um. The vessel density in the superficial capillary plexus (SCP) and the deep capillary plexus (DCP) was quantified automatically by AngioVue Analytics (RTVue-XR version 2017.1.0.155 software) [16]. The area, acircularity index (AI) and perimeter (PERIM) of fovea avascular zoon(FAZ), and the vessel density of the full retina in a width of 300 μm around the FAZ (FD-300) were also obtained by the software in the FAZ mode. The motion arteacts of eyes were decreased by eye tracking mode, and were removed by motion correction technology. The cutoff value of the signal strength index was set at ≥ 40.

### Statistical analysis

Statistical analysis was performed using SPSS software (version 21.0; IBM Corp.). Values were expressed as the mean ± standard deviation. BCVA was converted to Logarithm of the Minimum Angle of Resolution scoring.LSD-*t* test was used to compare the multiple means of visual acuity among the three groups.Differences in vessel density, FAZ, and AI between eyes with DRIL and without DRIL were tested by two independent-samples *t* test. Fisher’s exact test was used to test the difference between qualitative variables. The Spearman correlation coefficient and the multiple linear regression analysis were used to assess correlations. A value of *P* < 0.05 was accepted as statistically significant.

## Results

### Patient characteristics

74 eyes from 74 patients(age: 65.88 ± 5.66, 35 males and 39 females) which diagnosed with IERM were defined as eligible for this study. According to the superficial hemorrhage during the stripping operation, 35 patients in group A (no bleeding), 22 patients in group B (parafoveal bleeding) and 17 patients (macular fovea bleeding) in group C.There were no significant differences in age, course of disease, axial length, follow-up time, and CRT-1 mm at the time points of before and 12 months after surgery among the three groups (Table 1).

**Table 1:** Baseline characteristics of 74 eyes from 74 patients with IERM

	Group A	Group B	Group C	F	*P*
Patients	35	22	17		
Age (years)	64.80 ± 5.50	67.53 ± 5.02	65.81 ± 6.27	2.258	0.110
Disease duration (months)	5.83 ± 2.44	6.81 ± 3.13	6.84 ± 2.77	1.768	0.176
Axial length of eye (mm)	24.14 ± 0.90	23.96 ± 0.97	23.85 ± 0.95	0.928	0.399
Pre-op CRT-1 mm (um)	425.60 ± 22.22	417.97 ± 18.18	421.26 ± 22.64	1.264	0.287
Post-op CRT-1 mm (um)	345.45 ± 11.51	347.53 ± 9.91	348.58 ± 11.25	0.825	0.441

*IERM* idiopathic epiretinal membrane, *BCVA* best-corrected visual acuity, *Pre-op* pre-operative, *Post-op* post-operative, *CRT* central macular thickness

### Preoperative and postoperative BCVA

There were significant differences in the mean postoperative BCVA (logMAR) at 1, 3, 6 and 12 months among the three groups (*P* < 0.001). Specifically, in group C, the postoperative BCVA (logMAR) at 1, 3, 6 and 12 months was significantly higher than both Group A and Group B (*P* < 0.001). Moreover, the difference of post-operative BCVA (logMAR) between group A and group B was only appeared at 3 months after surgery (*p* = 0.004). The preoperative BCVA (logMAR) showed no difference among the three groups (F = 1.339, *P* = 0.267) (Table 2). Spearman correlation indicated that superficial hemorrhage was significantly correlated with poor postoperative BCVA (*r* = 0.698, *P = *0.000).

**Table 2:** Comparison of pre- and post operative BCVA in the three groups of 74 patients with IERM

	Pre-op	1 m Post-op	3 m Post-op	6 m Post-op	12 m Post-op
Group A	0.60 ± 0.16	0.40 ± 0.09	0.24 ± 0.08	0.16 ± 0.08	0.11 ± 0.09
Group B	0.59 ± 0.17	0.41 ± 0.10	0.31 ± 0.07	0.20 ± 0.09	0.14 ± 0.12
Group C	0.65 ± 0.16	0.79 ± 0.15	0.72 ± 0.13	0.66 ± 0.14	0.58 ± 0.17
F Value	1.339	131.838	262.623	258.310	147.316
*P*	*P* = 0.267	*P* < 0.001	*P* < 0.001	*P* < 0.001	*P* < 0.001
Group A&B	*P* > 0.05	*P* > 0.05	*P* = 0.004	*P* > 0.05	*P* > 0.05
Group B&C	*P* > 0.05	*P* < 0.001	*P* < 0.001	*P* < 0.001	*P* < 0.001
Group A&C	*P* > 0.05	*P* < 0.001	*P* < 0.001	*P* < 0.001	*P* < 0.001

### DRIL measurements

Among the 36 patients with DRIL by preoperative OCT, 24 patients had intraoperative superficial retinal hemorrhage (parafoveal: 10 cases, macular foveal: 14 cases) and that occurred in 15 of 38 patients (parafoveal: 12cases, macular foveal: 3 cases) without DRIL (*r* = 0.328, *P* = 0.000).

4 patients exhibited improved DRIL 1 month after operation. Furthermore, 13 additional patients demonstrated improved DRIL at 6 months post-operatively and 5 additional patients exhibited improvement at 12 months post-operatively. The mean pre-operative BCVA (logMAR) of patients without DRIL and with DRIL were (0.57 ± 0.21) and (0.14 ± 0.14), respectively (*r = *0.692, *P* = 0.000) (Table 3).

**Table 3:** Comparison of intraoperative status and postoperative BCVA in 74 IERM patients with or without DRIL

	Pre-op		12 m Post-op	
	With DRIL	Without DRIL	With DRIL	Without DRIL
Group A	12	23	1	34
Group B	10	12	2	20
Group C	14	3	11	6
*r*	0.328		0.547	
*P*	0.000		0.000	
BCVA	0.62 ± 0.16	0.58 ± 0.16	0.57 ± 0.21	0.14 ± 0.14
*r*	0.139		0.692	
*P*	0.149		0.000	

**Fig. 1:**
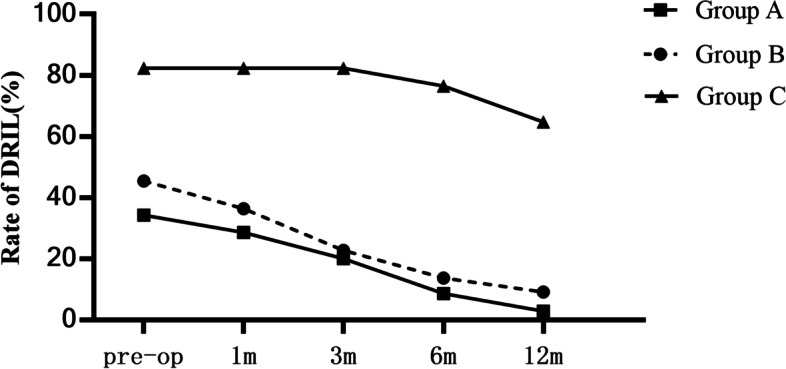
Pre- and post-operative changes in DRIL rate of IERM patients. Group A (35 cases). There were 12 cases with DRIL before operation, 10 cases had DRIL at post-op 1 m,7 cases at post-op 3 m, 3cases at post-op 6 m and only 1 case had DRIL left at post-op 12 m. Group B (22 cases). 10 cases with DRIL at pre-op, 8 cases at post-op 1 m, 5 cases at post-op 3 m,3 cases at post-op 6 m and only 2 cases at post-op 12 m. Group C (17 cases). 14 cases with DRIL at pre-op, 1 case showed improvement at post-op 6 m and 11 cases had DRIL left at post-op 12 m

**Fig. 2:**
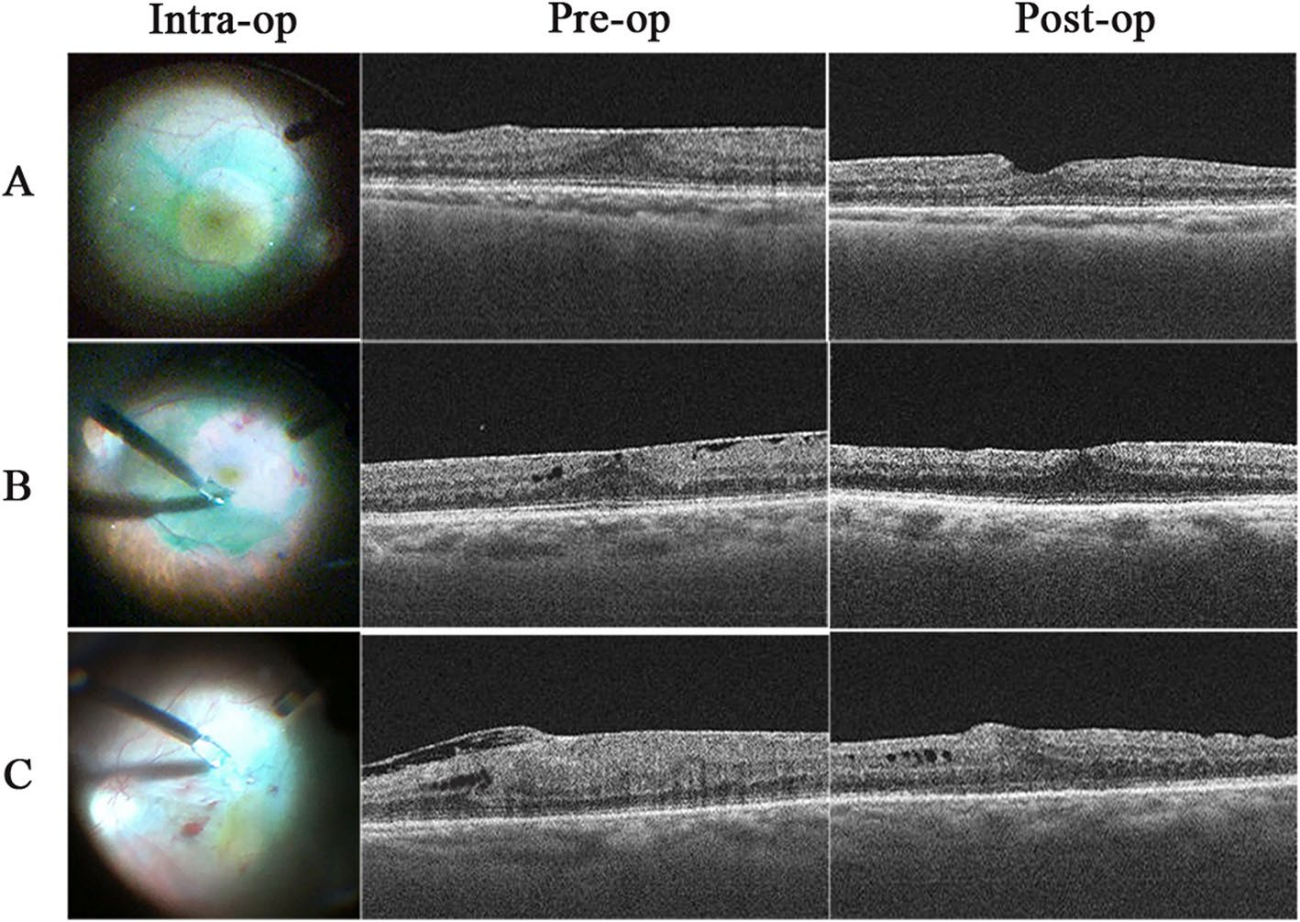
Intra-operative photos and OCT at pre-operative stage and 12 months post-operatively of patients with IERM. A. The patient with no hemorrhage during membrane peeling from Group A (male, 66 years old), which showed clear and identifiable inner retinal layer structure at pre-op and 12 m post-op. B. The patient with parafoveal bleeding during membrane peeling from Group B (female, 72 years old), which displayed DRIL at pre-op and improved at 12 m post-op. C. The patient with macular fovea bleeding during membrane peeling from Group C (female, 65 years old), which exhibited DRIL at pre-op and failed to recover at 12 m post-op

In group A, there were 12 cases with DRIL before operation, among which 9 cases had clear and identifiable inner retinal layer structure at 6 months after operation, and only 1 case had DRIL left at 12 months after operation. In group B, there were 10 cases with DRIL before operation, only 3 cases of inner retinal structure failed to recover at 6 months after operation, and only 2 cases with DRIL at 12 months after operation. In group C, 14 cases with DRIL before operation, 13 cases at 6 months after operation, and 11 cases left at 12 months after operation (Fig. 1, Fig. 2). The DRIL rates before surgery, and 1, 3, 6, and 12 months after surgery of the three groups were statistically significan (*r *= 0.328, *P* = 0.000) (*r* = 0.375, *P* = 0.000) (*r* = 0.457, *P* = 0.000) (*r* = 0.570, *P* = 0.000) (*r* = 0.589, *P* = 0.000).

### OCTA findings

**Fig. 3:**
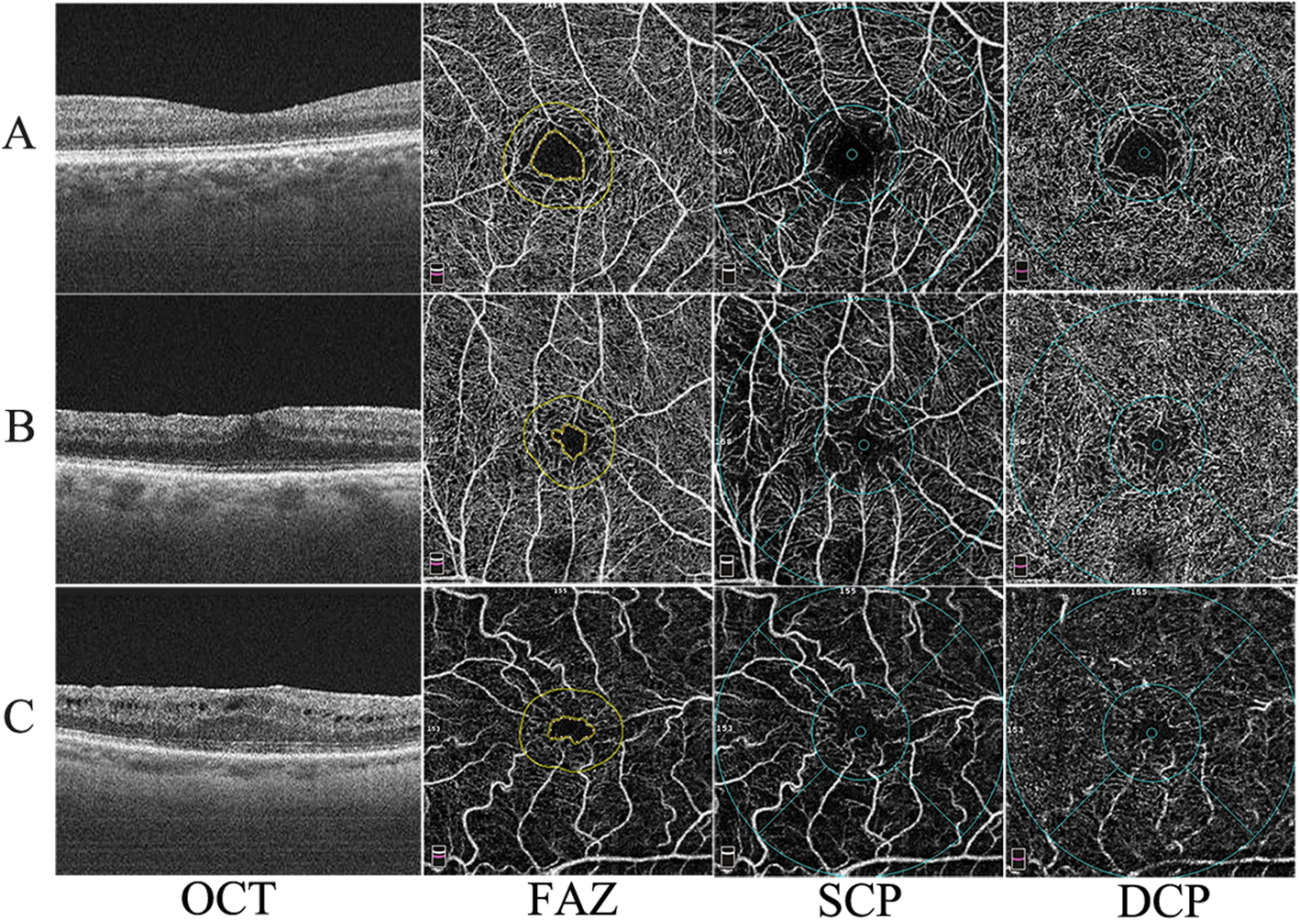
Post-operative OCT and OCTA images of control and IERM patients. A.OCT and OCTA images of a control patient (female, 53 years old), demonstrated normal reference structure of retinal, FAZ, SCP and DCP. B. OCT and OCTA images of an IERM patient (female, 65 years old) without DRIL at post-op12 months, had clear and identifiable inner retinal layer structure and a reduced area of FAZ with irregular morphology and tortuous vessel. C.OCT and OCTA images of an IERM patient (male, 61 years old) with DRIL at post-op12 months, showed inability to identify boundaries between the ganglion cell-inner plexiform layer, inner nuclear layer and outer plexiform layer and a typical ischemic appearance in both FD-300 (the area around the actual FAZ within 300 μm width marked yellow) and DCP, capillary network

OCTA was evaluated in 36 patients at 12 months post-operatively, including 14 eyes with DRIL and 22 eyes without DRIL. In all cases, OCT-A images showed a typical ischemic appearance in both SCP and DCP, deficient capillary network (Fig. 3). Quantitatively, OCT-A imaging revealed a significant decrease in vessel density in DCP in all parafoveal regions (superior: t = 3.795, *P* = 0.001; inferior: t = 3.390, *P* = 0.002;Nasal: t = 2.601, *P* = 0.014;Tempo: t = 3.106, *P* = 0.004) and FD-300 areas (t = 3.581, *P* = 0.001) in DRIL ( +) eyes compared with those in DRIL (-) eyes.(Table 4) However, there were no difference of FAZ,VD of SCP and DCP in macular fovea and SCP in parafovea in DRIL ( +) eyes compared with those in DRIL (-) eyes (Tables 5 and 6). The results of multiple linear regression indicated that only FD-300 was significantly correlated with postoperative BCVA (t = -2.807, *P* = 0.011).

**Table 4:** Comparison of FAZ measurements in 36 patients with or without DRIL

	FAZ area (mm^2^)	PERIM (mm)	AI	FD (%)
Without DRIL	0.11 ± 0.04	1.42 ± 0.28	1.21 ± 0.05	45.08 ± 3.71
With DRIL	0.10 ± 0.03	1.32 ± 0.10	1.18 ± 0.06	40.27 ± 4.26
t	0.071	1.472	1.272	3.581
*P*	0.944	0.152	0.212	0.001

*FAZ* fovea avascular zone, *PERIM* FAZ perimeter, *AI* A-circularity index, *FD* foveal vessel density in a 300 μm wide region around FAZ, *DRIL* disorganization of retinal inner layers

**Table 5:** Comparison of superficial capillary plexus (SCP) measurements in 36 patients with or without DRIL

	Fovea VD (%)	Tempo VD (%)	Superior VD (%)	Nasal VD (%)	Inferior VD (%)
Without DRIL	28.63 ± 5.72	39.01 ± 3.87	39.50 ± 4.09	40.25 ± 3.81	40.13 ± 4.84
WithDRIL	25.97 ± 4.37	39.10 ± 2.31	39.13 ± 2.73	38.70 ± 2.32	39.21 ± 2.83
t	1.484	-0.075	0.296	1.364	0.637
*P*	0.147	0.941	0.769	0.182	0.528

**Table 6:** Comparison of deep capillary plexus (DCP) measurements in 36 patients without or with DRIL

	Fovea VD (%)	Tempo VD (%)	Superior VD (%)	Nasal VD (%)	Inferior VD (%)
Without DRIL	43.15 ± 5.89	49.40 ± 3.46	50.10 ± 3.07	49.53 ± 3.49	49.84 ± 2.42
With DRIL	41.43 ± 3.64	44.99 ± 5.08	45.01 ± 5.00	46.36 ± 3.66	46.76 ± 2.99
t	0.978	3.106	3.795	2.601	3.390
*P*	0.335	0.004	0.001	0.014	0.002

## Discussion

Pars plana vitrectomy (PPV) with membrane peeling is the standard treatment to release the forces and restore the normal structure of the macula of IERM eyes. Some surgeons consider ILM peeling as an important aid in the removal of residual ERM [4], because of a lower recurrence rate [5]. On the other hand, ILM peeling has been shown to cause structural damages of retina [17]. The effects of IERM operations on postoperative visual acuity were investigated, including the difficulty of stripping, indocyanine green staining and intraoperative bleeding. Multivariate Logistic regression analysis showed that foveal retinal surface bleeding during stripping was an important factor leading to postoperative poor visual acuity [6]. However, the reason is still unclear.

To our best knowledge, this is the first study to investigate that superficial hemorrhage after membrane peeling is associated with DRIL based on OCT data of 74 IERM patients, and the corresponding changes of macular morphology and blood flow density in these patients were observed by OCTA to quantitatively analyze the retinal blood vessels in patients with persistent DRIL after IERM, so as to clarify the reason why the foveal hemorrhage during IERM surgery seriously affects the postoperative visual recovery of patients.

Although a large number of studies have focused on changes in microstructure of fovea due to IERM to identify the anatomic changes associated with the vision recovery after surgery, the predictors of postoperative visual outcome are still under investigated [18].DRIL was first detected in OCT imaging in 2014 [7]. It is believed that the DRIL indicates retinal vascular dysfunction and the damage of retinal microcirculation that will lead to destruction of neural structure [15]. Therefore, DRIL can predict the collapse of anatomical structures in the visual transmission pathway [19]. Nicholson suggested that DRIL could be used as an OCT biomarker to predict BCVA in DME patients [15]. Radwan and associates studied the correlation between the length of DRIL and visual changes [9]. Similarly, several studies have shown that the inner retinal layer is important for assessing visual prognosis in IERM patients [14, 20, 21]. Accordingly, DRIL has been gradually recognized as a "marker" of visual prognosis in patients with macular disease [2–9].

As the first study to correlate retinal nerve fiber layer bleeding during ILM peeling with DRIL,it was found that the probability of intraoperative bleeding in patients with preoperative DRIL was significantly higher than that in patients without preoperative DRIL especially in macular foveal. It was further found that the recovery rate of DRIL in patients with macular foveal bleeding was only 21.43% (3/14), which was significantly lower than that in patients without bleeding (91.67%, 11/12). Therefore, we concluded that DRIL is one of the causes of retinal bleeding in IERM surgeries. The possible pathological mechanism is that the mechanical traction force of IERM not only destroys the normal macular capillary distribution and also is accompanied by various nerve cell damage, macular capillary ischemia and microcirculation destruction [22], resulting in abnormal distribution and exudation of blood vessels, extracellular fluid accumulation in the inner retinal space, and finally the inner retinal layer structure disorder. Macular vascular abnormalities cause bleeding during IERM sugery, and hemorrhagic injury further aggravate the destruction of macular microcirculation, which is responsible for the persistence of DRIL and poor postoperative visual recovery. Therefore, preoperative evaluation of DRIL in IERM patients will be helpful to predict their postoperative visual acuity.

At present, there are limited studies on DRIL and impaired retinal microcirculation [23]. Some studies have found that the vascular density in the foveal region of DRIL patients is significantly decreased in diabetic retinopathy, suggesting that DRIL and the abnormal blood perfusion in the macular foveal region interact with each other [24]. OCTA were frequently used to study the changes in macular microvascular characteristics, and FD-300 is a foveal vessel density automatically identified by OCTA around the actual FAZ area within 300 μm width. Recent studies have reported that FD-300 index can accurately monitor early diabetic retinopathy [25], and can determine the prognosis and treatment response of DME [25, 26]. In this study, retinal microvascular quantitative analysis was performed in 36 IERM patients with (14 eyes) or without (22 eyes) DRIL. It was found that the patients with DRIL had no significant difference in the VD of DCP and SCP in macular fovea, but FD-300 was significantly decreased. Compared with foveal DCP and SCP, FD-300 can minimize the bias caused by FAZ morphology and retinal stratification in the evaluation of foveal microvessel density [25]. Previous studies suggested that the integrity of microvasculature around the FAZ may play a critical role in the homeostasis of fluid [27]. In this study, FD-300 was used to evaluate the changes of macular foveal vessel density in IERM patients for the first time. Lower FD-300 showed more severe damage in juxtafoveal capillaries and worse microangiopathy. Multiple linear regression analysis showed that FD-300 can predict the post-operative visual acuity of IERM patients. Therefore, FD-300 deserves further attention as a reference index for postoperative visual acuity in patients with IERM.

In the analysis of parafoveal VD, the difference between the patients with and without DRIL after IERM surgery was mainly found in VD of DCP. This result is consistent with the previous study, which found that focal perfusion defects identified by FFA corresponded to the locations of absent or low flow signals in DCP, however, no sign of non-perfusion was observed in these locations in distorted SCP vessels [28]. Lin and associates suggested that mechanical stress caused by ERM more profoundly affects the DCP than the SCP in regard to decrease in blood flow [29]. In addition, multistage capillary non-perfusion may be one of the important pathogenesis of DRIL [30]. The reason why DCP is more prone to damage may be related to its anatomical tissue characteristics. In addition, while the recovery of defects were observed in some eyes, the damage of some others could not be reversible due to focal non-perfused areas affecting the function of the inner retinal layer. Therefore, we speculate that the abnormal deep capillaries in IERM patients hinder the repair of postoperative DRIL, and the persistent DRIL is an important indicator for postsurgical visual prognosis.

In order to eliminate the interference, patient selection was as strictly as possible in this study. Patients with diabetes mellitus, high myopia, uveitis, systemic diseases, incomplete outer retinal structure and severe macular edema (CFT-1 mm > 363um) on OCT were excluded, to focus on the inner retina solely. In addition, considering the average age of the 74 patients (65.55 years old) and the impact of the operation on the lens, all patients were perfomed cataract extraction combined with intraocular lens implantation.The baseline visual acuity of 74 patients with IERM was not correlated with preoperative DRIL, and preoperative lens status may be one reason.

There are limitations to our study. First, since this study was retrospective, some of the results should be treated with caution, and future prospective studies are needed. Second, not all patients conducted OCT-A because of some patients with good visual recovery lost to follow-up. In addition, because of the complexity of its pathogenesis and the diversity of its pathogenic course, it is difficult to accurately predict the prognosis of IERM.

## Conclusions

Our study preliminary established that the DRIL predicted superficial hemorrhage during membrane peeling, and the bleeding was a reason for the problematic recovery of the DRIL in turn. The persistence of DRIL directly affects the postoperative vision of patients with IERM. Moreover, our results indicated that OCT-A could precisely quantify the reduction in capillary plexus perfusion in IERM patients with DRIL. Therefore, DRIL is a novel valuable index for evaluating intraoperative situations and postoperative vision in IERM patients. This should be considered in the decision process whether to perform ERM peeling in this patient group.

## Data Availability

The datasets used and analysed during the current study are available from the corresponding author on reasonable request.

## Abbreviations

IERM: Idiopathic epiretinal membrane
DRIL: Retinal inner layers
OCTA: Optical coherence tomography angiography
OCT: Optical coherence tomography
BCVA: Best-corrected visual acuity
SCP: Superficial capillary plexus
DCP: Deep capillary plexus
AI: Acircularity index
FAZ: Fovea avascular zoon
PPV: Pars plana vitrectomy
ILM: Internal limiting membrane
DR: Diabetic retinopathy
RVO: Retinal vein occlusion
Pre-op: Pre-operative
Post-op: Post operative
CRT: Central macular thickness
FD: Foveal vessel density
PPV: Pars plana vitrectomy
